# IGBT Gate Boost Drive Technology for Promoting the Overload Capacity of Traction Converter

**DOI:** 10.3390/mi15060738

**Published:** 2024-05-31

**Authors:** Yunxin Zhang, Xiaodong Dong, Linxia Wu, Xiaoyu Wang, Ming Ma, Xianjin Huang, Yong Jin, Pengze Zhu

**Affiliations:** 1 CECEP Wind-Power Corporation Co., Ltd., Beijing 100089, China; zhangyunxin@cecwpc.cn (Y.Z.); dongxiaodong@cecwpc.cn (X.D.); 2 CECEP (Inner Mongolia) Wind-Power Corporation Co., Ltd., Baotou 014500, China; wulinxiay@cecwpc.cn (L.W.); wangxiaoyu@cecwpc.cn (X.W.); 3 CECEP (Xinjiang) Wind-Power Corporation Co., Ltd., Urumqi 830063, China; chenqixiang@cecwpc.cn; 4 School of Electrical Engineering, Beijing Jiaotong University, Beijing 100044, China; 23111446@bjtu.edu.cn (Y.J.); 23126409@bjtu.edu.cn (P.Z.)

**Keywords:** IGBT drive circuit, gate voltage boosting technology, temperature rise, IGBT loss, traction converter

## Abstract

Under certain circumstances, a high-speed railway may require constant acceleration or emergency braking, in which case the inverter may experience short-term overload conditions and the current passing through the IGBT will go beyond the rated design tolerance. Under overload conditions, the IGBT loss will increase instantly, raising the power semiconductor device’s junction temperature in the process. This research examines the boosting-gate-voltage-driven IGBT control technology. It increases the gate drive voltage and the IGBT current capacity and decreases the conduction voltage drop of IGBT under short-term overload conditions, reducing the instantaneous loss and temperature rise undulation of IGBT. The working characteristics of IGBT devices are studied, and the influence of gate drive voltage on device loss and temperature rise fluctuations is analyzed. Based on the emergency acceleration and brake conditions of the actual train operation, the short-term overload characteristics of the inverter are analyzed. The optimization analysis of the boosting gate voltage under emergency conditions is carried out, and the IGBT drive circuit with gate voltage pumping function is designed. The effectiveness of the driving circuit is verified through PSpice simulation and actual switching characteristic test. According to the analysis of experimental data, it can be verified that increasing the gate voltage technology can reduce IGBT losses.

## 1. Introduction

In China’s rail transit vehicles, AC traction has become the most important transmission system. With the advantages of low on-impedance, low driving power consumption and high withstand voltage level, the IGBT device has emerged as one of the mainstays of traction drive systems [1,2,3]. There is a certain amount of power transfer loss when the traction converter operates normally. IGBT devices are responsible for about 90% of the overall loss, and these losses are converted into heat. Electric loss causes the working junction temperature of IGBTs to increase, and high temperatures alter the properties of power devices without causing damage to the devices themselves. Device aging is accelerated by junction temperature changes and influences that affect power module packaging layer soldering fatigue. The device’s good functioning state can be maintained by maintaining a steady operating junction temperature. Increasing the current capability of the IGBT device or designing sufficient current headroom can reduce temperature rise fluctuations due to device losses. IGBT loss and heat dissipation are the main factors affecting the temperature rise of devices and converters. In [4,5], the cooling system’s thermal resistance can be changed to regulate the IGBT junction temperature. This approach requires no new equipment and has no effect on the original control strategy. Nevertheless, the heatsink has a considerable heat capacity, therefore, real-time temperature adjustment is not possible.

Reduction in IGBT loss, primarily through modulation, system control, and gate drive, is frequently used to control IGBT temperature rise. Several modulation techniques are employed in [6,7] to regulate the IGBT switching sequence and lower the loss of IGBT. However, this technique will produce waveforms with poor quality. In [8,9,10], the system control method is adopted. By optimizing the running curve of the train, the loss of IGBT in the whole process is minimized. However, this method is only suitable for the normal working conditions and cannot control the temperature rise in emergency conditions in real time. The gate drive circuit is the medium of control system and power system. It can control the performance of IGBT by adjusting the relevant parameters, so as to achieve the purpose of regulating the loss of IGBT. In [11,12], a strategy that is driven hierarchically is used. IGBT switching is broken down into steps, with varying gate resistances being employed at each stage. Lower switching losses can be attained. But the detecting and switching circuit is overly intricate, making the design more challenging. In [13,14], a scheme to change the gate voltage is proposed. The switching process and switching losses are optimized. However, the gate voltage of 15 V is still maintained after the IGBT is turned on, and the conduction loss remains unchanged. The gate drive technology in the above literature only considers the characteristics of IGBT itself. Few literatures optimize the performance of IGBT from the system level in combination with the train working conditions.

For a brief while, the traction converter will be overwhelmed when the train is braking or in acceleration in an emergency. The current passing through the IGBT will now increase. IGBT devices with a higher current capacity or parallel connection are selected for the traction converter design. The safety working margin of the existing IGBT devices has been reserved relative to the parameter manual. Due to the acceleration of the train, the IGBT current increases, but the capability of the device itself is not fully utilized, resulting in a certain capacity waste. So, a method to boost the IGBT gate voltage is proposed, which can improve the short-term current passing capability. To guarantee the train’s dependable operation, the gate voltage is raised, which also lowers the IGBT’s loss and temperature rise.

## 2. Characteristics of Traction Inverter

### 2.1. Analysis of Traction Converter System

**Figure 1:**
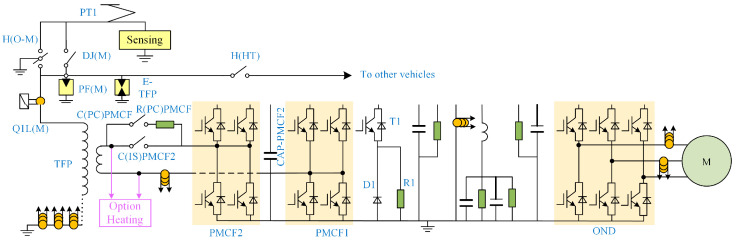
Circuit diagram of traction electric drive system.

Figure 1 displays the traction electric drive system’s primary circuit diagram. The traction transformer, traction converter, and traction motor make up this system [15]. The traction transformer receives AC power from the catenary to provide power for the whole train. The traction motor converts electric energy to kinetic energy to drive the whole train forward. Traction converter is the core part of this system, which is mainly composed of four quadrant rectifiers, an over-voltage suppression circuit and a traction inverter. Different circuits have different functions, but their cores are IGBT modules.

The traction converter is mainly composed of IGBT module. The four-quadrant rectifier is composed of PMCF1 and PMCF2. The overvoltage suppression circuit is composed of T1, D1 and R1. The traction inverter is composed of six IGBT modules, as shown in the OND part in Figure 1.

Different working conditions of the train are mainly related to the status of traction motor. The traction inverter has the closest relationship with the traction motor. By controlling the switching state of IGBT, different power can be realized to drive the traction motor. Therefore, the traction inverter is selected as the research object in this paper.

**Figure 2:**
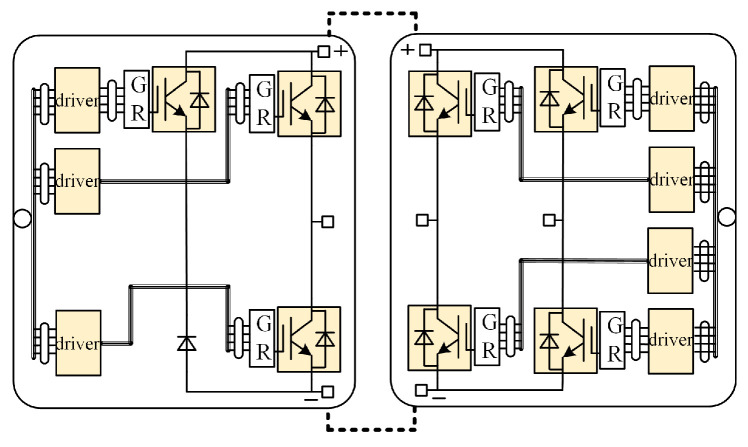
The connection of the IGBT module and the driver circuit.

Figure 2 shows the connection of the IGBT module and the driver circuit. In addition to the three bridge arms of the inverter, it also includes overvoltage suppression circuit. GR represents the gate resistance adapter board, which is directly connected to the gate of IGBT to reduce parasitic parameters. The IGBT drive circuit is connected with the gate resistance adapter board through wires, which is convenient for the flexible layout of the drive circuit.

### 2.2. Overload Condition of Traction Inverter

**Figure 3:**
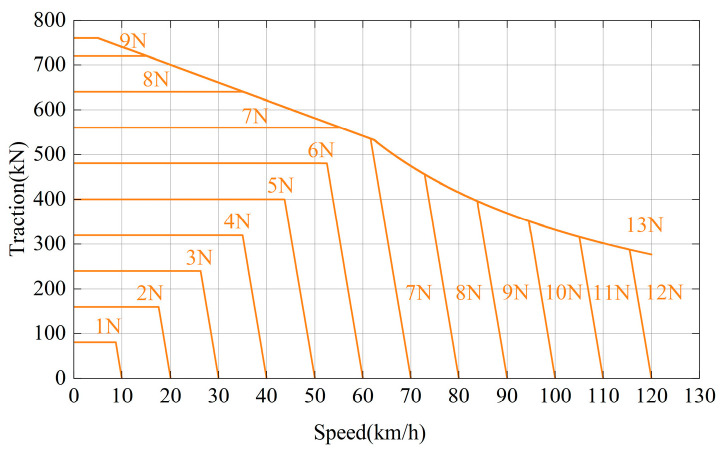
Traction characteristic curve.

The working principle of traction inverter is basically the same, but the working conditions vary greatly with the different routes. As shown in Figure 3, the traction characteristic curve of the HXD2 electric locomotive is given, and the traction characteristic curve of the control handle in different gears N is marked in this figure [16]. The gears N of the train are divided into 13 levels, the curve of which corresponding to the 13-level gear is the maximum traction characteristic curve of the traction converter.

The HXD2 electric locomotive adopts a quasi-constant speed control strategy. When the speed is small, the traction force remains constant and the train is in the traction condition. When the speed reaches a certain value, the traction force automatically drops to around 0. The train keeps running at a constant speed, the traction force is equal to the basic resistance. At this time, the train is in the cruising condition.

In order to accurately express the traction characteristics of the train, a mathematical model is established for the curve shown in Figure 3:

(1) $$F_{1}=80n$$

(2) $$F_{2}=640n-64v$$

(3) $$F_{3}=\left\{\begin{array}{ll} 760 & v<5\\ 779.7-3.97\times v & 5\leq v<62.4\\ 33,200/v & v\geq 62.4 \end{array}\right.$$

(4) $$F=\min\left\{F_{1},F_{2},F_{3}\right\}$$

*F*_1_ determines the traction force in the horizontal section of the traction characteristic curve, *F*_2_ determines the traction force in the linear change phase; *F*_3_ determines the maximum traction force. *F* represents the total traction force of the train, taking the minimum value of *F*_1_, *F*_2_, and *F*_3_, and the unit is kN. *n* is the level of the control handle, and *v* is the speed of the train; the unit is km/h.

According to different environments and road conditions, the train driver can control the gear to make the train work on different traction characteristic curves. The active power at the output side of traction motor can be obtained by multiplying the total traction force of the train with its speed. The apparent power at the input side of traction motor *S*_in_ can be obtained by dividing the power by the transmission efficiency *η* and power factor cos *φ*:

(5) $$S_{\mathrm{in}}=\frac{Fv}{3.6\eta \cos\;\varphi }$$

The voltage of DC side of the traction inverter remains unchanged at 1800 V, and the output voltage RMS of the inverter is: $U_{o}=\sqrt{6}/\pi \times U_{\mathrm{dc}}$. Therefore, the current RMS value on the output side of the inverter *I*_0_ is:

(6) $$I_{o}=\frac{S_{\mathrm{in}}}{\sqrt{3}U_{o}}=\frac{Fv}{\frac{54\sqrt{2}}{5\pi }\eta U_{\mathrm{dc}}\cos\;\varphi }$$

*v* is the speed of the train, *η* is the traction motor efficiency, cos *φ* is the power factor of traction motor, and *U*_dc_ is the input voltage of the traction inverter. The IGBT collector current is positively correlated with the output current RMS of traction inverter.

The working conditions of train are changing all the time. When passing through some special road sections, the train needs to speed up as soon as possible. At this time, the traction inverter needs to output a large amount of power to obtain sufficient traction force. When the train runs on a small ramp, it is necessary to increase the traction force of the train in time to speed up. The speed of the train should be increased as much as possible to save enough kinetic energy for the train to ensure that the train passes through difficult sections.

Take the train emergency traction condition as an example. When the train is running smoothly, the control handle is in the 8-level, and the train speed is 80 km/h. In order to pass through the difficult section as soon as possible, the driver pushes the control handle to the 10-level to obtain greater traction force and increase the speed of the train.

The train is in the cruising condition when it maintains a speed of 80 km/h. The train’s traction force and the fundamental resistance are now equal. As per [17], the train’s fundamental unit resistance is:

(7) $$\omega_{0}=0.84+0.0012v+0.000313v^{2}$$

The traction mass multiplied by the basic unit resistance determines the train’s basic resistance:

(8) $$W_{0}=\omega_{0}M$$

The traction mass *M* is 10,000 t. At 80 km/h, the resistance of the train is 29.4 kN. Since the train has four traction motors, the traction force of each motor is about 7.35 kN. According to (6), the output current RMS of the traction inverter is *I*_o_ = 79.8 A.

When the control handle is adjusted to level 10, the train is in the traction condition. At this time, *n* = 10, *v* = 80 km/h, *F*_1_ = 800 kN, *F*_2_ = 1280 kN, *F*_3_ = 415 kN. Taking the minimum value of *F*_1_, *F*_2_ and *F*_3_, the traction force *F* of the train is 415 kN. Therefore, the traction force of each motor is about 103.75 kN. According to (6), the output current RMS of the traction inverter is *I*_o_ = 1126.1 A. However, the rated current of the traction inverter is only 460 A. Therefore, when the train is under emergency traction condition, the traction inverter works in the overload condition.

In overload conditions, the current of the traction inverter increases suddenly. This working condition needs to be considered in the design of the converter system to improve the reliability of the entire system.

## 3. Analysis of IGBT Temperature Rise and Working Characteristics

### 3.1. Analysis of IGBT Temperature Rise

The temperature of IGBT is mainly related to the loss and heat dissipation [18]. When the traction inverter works in an overload condition, the loss of IGBT will increase sharply. Under the same heat dissipation conditions, IGBT will have large temperature fluctuation.

In the electric traction system, the loss comes from the IGBT module. An IGBT module consists of an IGBT and a diode. The IGBT loss *P*_I_ includes the on-state loss *P*_I-on_ and the switching loss *P*_I-s_. The diode loss *P*_D_ includes the diode on-state loss *P*_D-on_ and the turn-off loss *P*_D-s_. In order to calculate the loss of IGBT more accurately, the characteristic curve of IGBT is divided into two sections [19]. At the same time, the bipolar modulation strategy is adopted, and the calculation formula of the correlation loss can be finally obtained. 

The IGBT on-state loss is:

(9) $$\left\{\begin{array}{cc} \begin{array}{c} P_{I\_\mathrm{on}}=(\frac{1}{2\pi }+\frac{M\cos\;\varphi }{8})\frac{1}{2}V_{\mathrm{CEO}}I_{\mathrm{CP}}\\ +(\frac{1}{8}+\frac{M\cos\;\varphi }{3\pi })r_{\mathrm{CE}}^{\prime }I_{\mathrm{CP}}^{2} \end{array} & 0\leq I_{\mathrm{CP}}\leq \frac{1}{3}I_{\mathrm{CN}}\\ \begin{array}{c} P_{I\_\mathrm{on}}=(\frac{1}{2\pi }+\frac{M\cos\;\varphi }{8})V_{\mathrm{CEO}}I_{\mathrm{CP}}\\ +(\frac{1}{8}+\frac{M\cos\;\varphi }{3\pi })r_{CE}I_{\mathrm{CP}}^{2} \end{array} & I_{\mathrm{CP}}\geq \frac{1}{3}I_{\mathrm{CN}} \end{array}\right.$$

The on-state loss of the diode is:

(10) $$\left\{\begin{array}{cc} \begin{array}{c} P_{D\_\mathrm{on}}=(\frac{1}{2\pi }-\frac{M\cos\;\varphi }{8})\frac{1}{2}V_{\mathrm{FO}}I_{\mathrm{CP}}\\ +(\frac{1}{8}-\frac{M\cos\;\varphi }{3\pi })r_{F}^{\prime }I_{\mathrm{CP}}^{2} \end{array} & 0\leq I_{\mathrm{CP}}\leq \frac{1}{3}I_{\mathrm{CN}}\\ \begin{array}{c} P_{D\_\mathrm{on}}=(\frac{1}{2\pi }-\frac{M\cos\;\varphi }{8})V_{\mathrm{FO}}I_{\mathrm{CP}}\\ +(\frac{1}{8}-\frac{M\cos\;\varphi }{3\pi })r_{F}I_{\mathrm{CP}}^{2} \end{array} & I_{\mathrm{CP}}\geq \frac{1}{3}I_{\mathrm{CN}} \end{array}\right.$$

*I*_CP_ is the amplitude of inverter output current, which is 1.414 times of *I*_o_. *I*_CN_ is the rated current of IGBT, *M* is the modulation ratio, cos *φ* is the power factor, *V*_CEO_ is the intersection of the IGBT output characteristic curve after linearization and the horizontal axis. *r*’_CE_ is the on-state resistor of the IGBT current stage between 0 and *I*_CN_/3 and *r*_CE_ is the on-stage resistor of the IGBT current stage between *I*_CN_/3 and *I*_CP_. *V*_FO_ is the intersection of the diode output characteristic curve after linearization and the horizontal axis. *r*’_F_ is the on-state resistor of the diode current stage between 0 and *I*_CN_/3 and *r*_F_ is the on-stage resistor of the IGBT current stage between *I*_CN_/3 and *I*_CP_.

The switching loss of the IGBT is:

(11) $$\left\{\begin{array}{ll} \begin{array}{l} P_{I\_s}=\frac{1}{\pi }f_{\mathrm{sw}}\frac{V_{\mathrm{dc}}}{V_{\mathrm{CEN}}}[E_{\mathrm{on}}+E_{\mathrm{off}}\\ \;\;+\frac{3(I_{\mathrm{CP}}-I_{\mathrm{CN}})}{2I_{\mathrm{CN}}}(E_{\mathrm{on}}+E_{\mathrm{off}}-E_{\mathrm{on}\frac{1}{3}}-E_{\mathrm{off}\frac{1}{3}})] \end{array} & 0\leq I_{CP}\leq I_{CN}\\ \begin{array}{l} P_{I\_s}=\frac{1}{\pi }f_{\mathrm{sw}}\frac{V_{\mathrm{dc}}}{V_{\mathrm{CEN}}}[E_{\mathrm{on}}+E_{\mathrm{off}}\\ \;\;+\frac{3(I_{\mathrm{CP}}-I_{\mathrm{CN}})}{2I_{\mathrm{CN}}}(E_{\mathrm{on}\frac{5}{3}}+E_{\mathrm{off}\frac{5}{3}}-E_{\mathrm{on}}-E_{\mathrm{off}})] \end{array} & I_{CP}\geq I_{CN} \end{array}\right.$$

*f*_sw_ is the switching frequency, *V*_dc_ is the DC side voltage; *V*_CEN_ is the voltage across CE when the IGBT is turned off under rated conditions, *E*_on_ and *E*_off_ are the turn-on and turn-off energy at the rated current. *E*_on1/3_ and *E*_off1/3_ are turn-on and turn-off energy at 1/3 rated current; *E*_on5/3_ and *E*_off5/3_ are turn-on and turn-off energy at 5/3 rated current.

The diode turn-off loss is:

(12) $$P_{D\_s}=\frac{1}{\pi }f_{\mathrm{sw}}E_{\mathrm{Doff}}\frac{I_{\mathrm{CP}}}{I_{\mathrm{CN}}}\frac{V_{\mathrm{dc}}}{V_{\mathrm{CE}}}+VP_{\mathrm{Doff}}$$

(13) $$\left\{\begin{array}{ll} VP_{\mathrm{Doff}}=\frac{1}{5\pi }E_{\mathrm{Doff}}f_{\mathrm{sw}} & 0\leq I_{\mathrm{CP}}\leq \frac{1}{2}I_{\mathrm{CN}}\\ VP_{\mathrm{Doff}}=\frac{I_{\mathrm{CN}}-I_{\mathrm{CP}}}{8000\pi }f_{\mathrm{sw}} & \frac{1}{2}I_{\mathrm{CN}}\leq I_{\mathrm{CP}}\leq I_{\mathrm{CN}}\\ VP_{\mathrm{Doff}}=\frac{I_{\mathrm{CN}}-I_{\mathrm{CP}}}{6000\pi }f_{\mathrm{sw}} & I_{\mathrm{CP}}\geq I_{\mathrm{CN}} \end{array}\right.$$

*E*_Doff_ is the turn-off loss of the diode under rated conditions. The relevant data of the train under emergency traction conditions are shown in Table 1.

The power module of the traction inverter is Mitsubishi’s CM1200HC-66H 3300 V/1200 A IGBT module. According to the characteristic curve of its datasheet, the loss is calculated in Table 2.

The increase in temperature of IGBT is directly associated with the heat dissipation system in addition to its own loss. Three methods are often used by the traction inverter cooling system: heat pipe cooling, liquid cooling, and air cooling. Either heat dissipation method can be represented by a model of thermal resistor and thermal capacitor.

There are currently two main thermal network models: the Cauer model and the Foster model [20]. The Cauer model is a model directly established according to the actual physical layer and material layer of IGBT. But, it is difficult to obtain the parameters of this model. Although Foster has no actual physical meaning, the parameters of this model are easily obtained from actual measurements, so the model is more widely used.

**Figure 4:**
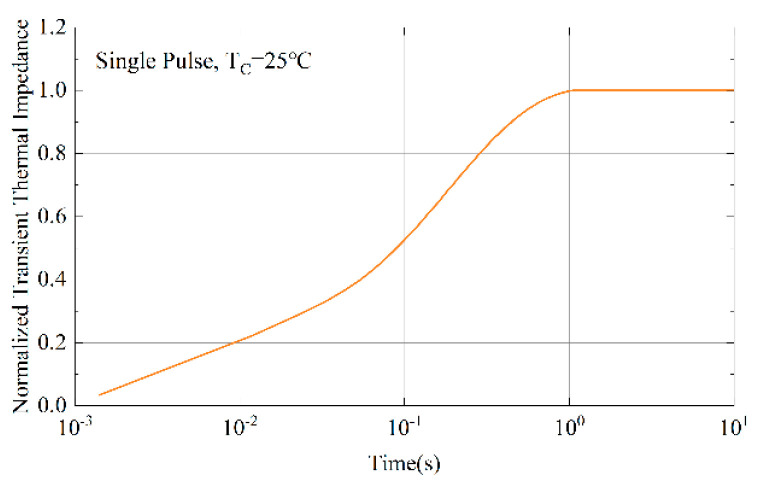
IGBT thermal impedance curve.

Figure 4 shows the thermal impedance test curve of CM1200HC-66H. After the power device is turned on for 1 s, the thermal impedance of IGBT basically reaches a steady state. Therefore, the steady-state thermal impedance value is taken when calculating the junction temperature of the power device in the traction inverter.

**Figure 5:**
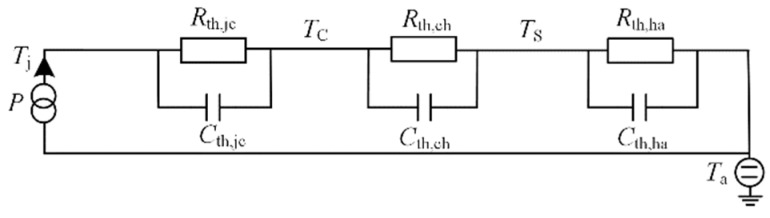
IGBT thermal network model.

According to Figure 5, the Foster thermal network model for the traction inverter is established without taking into account the uneven temperature distribution on the surface of the power device and the heatsink, where *T*_C_ is the case temperature of the IGBT, *T*_S_ is the surface temperature of the heatsink, *T*_j_ is the junction temperature of the IGBT, *T*_a_ is the ambient temperature, *R*_th,jc_ is the thermal resistor between the semiconductor junction and the case, *R*_th,ch_ is the thermal resistor between the case and the heatsink, and *R*_th,ha_ is the thermal resistor between the heatsink and the environment.

The junction temperature of IGBT is:

(14) $$T_{j}=P[R_{\mathrm{th},\mathrm{jc}}+R_{\mathrm{th},\mathrm{ch}}+R_{\mathrm{th},\mathrm{ha}}]+T_{a}$$

The calculation results are shown in Table 3. The data show that the temperature rise of the IGBT under the traction condition is greater than that under the cruise condition. The lifetime of IGBT is related to the temperature fluctuation. Therefore, the life of IGBT will be affected when the traction converter is under overload condition.

It is necessary to find a method to reduce the loss of IGBT, thereby reducing the temperature fluctuation of IGBT.

### 3.2. Analysis of IGBT Working Characteristics

Traction-grade IGBTs need to face more complex application environments, including power supply network voltage fluctuations, frequent load changes, high temperature and severe cold climates, and vibration environments [21]. Higher standards are established for IGBT performance in order to guarantee system dependability.

**Figure 6:**
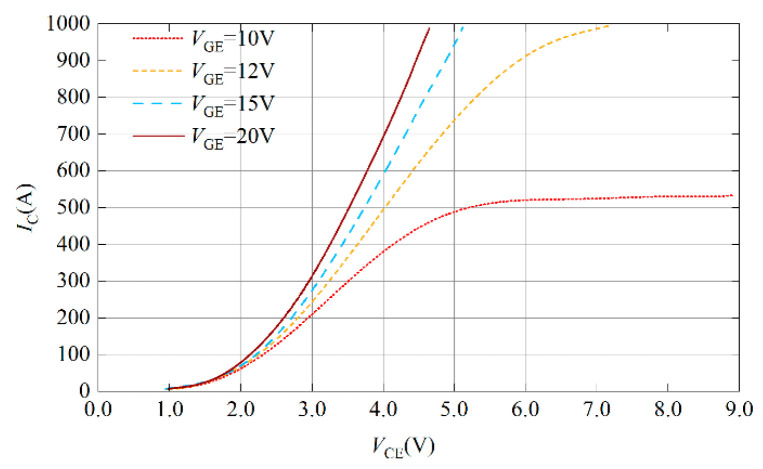
Output characteristics curve of FD500R65KE3.

As shown in Figure 6, the output characteristic curve of the traction-grade IGBT module FD500R65KE3 is given. The on-state voltage drop of IGBT gradually decreases with the increase in the gate voltage. Therefore, the loss of IGBT can be reduced by increasing the gate voltage.

**Figure 7:**
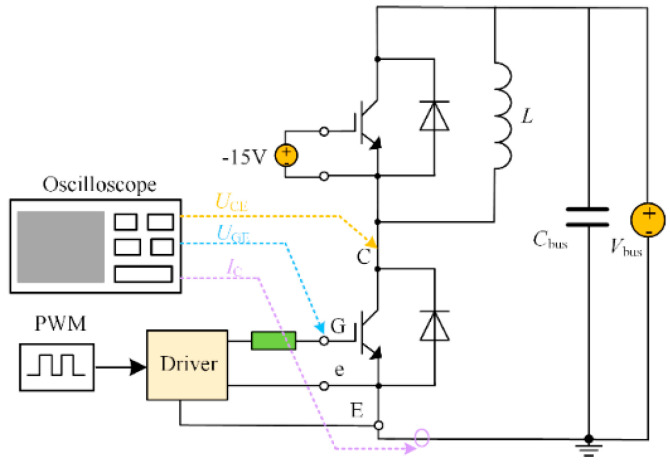
Switching characteristic test circuit.

**Figure 8:**
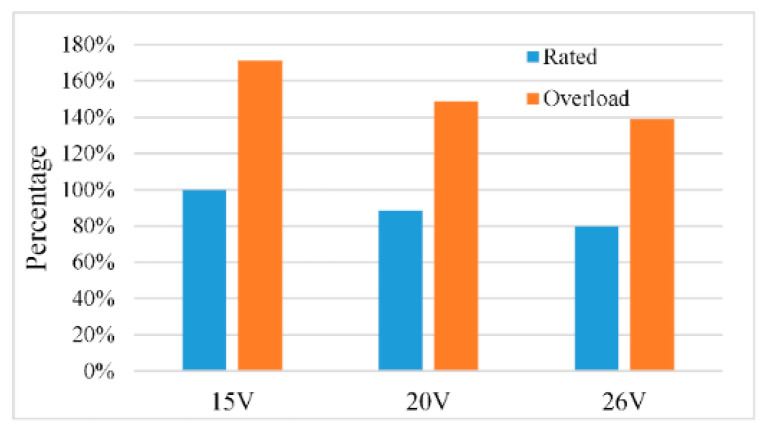
Variation diagram of IGBT loss under different gate voltages.

In order to verify the influence of gate voltage on the loss of IGBT, the switching characteristic test circuit shown in Figure 7 is built in PSpice simulation software. The relevant simulation parameters are shown in Table 4. IGBT operating circumstances are separated into two categories during the simulation: rated condition and overload condition. The collector current flowing through the IGBT is the rated value while operating under rated conditions. Under overload condition, the collector current passing through the IGBT is 1.5 times the rated value. The switching frequency of the IGBT is 800 Hz and the duty cycle is 50%. Figure 8 shows the loss change of the FD500R65KE3 under different gate voltages and different working conditions. The loss data of the IGBT is normalized, and the loss data under the rated working condition of the 15 V gate voltage is used as the reference value. The percentage change of IGBT loss under different working conditions is obtained. Figure 8 shows that under overload conditions, the IGBT loss will significantly increase; however, the IGBT loss can be decreased by raising the gate voltage.

The data show the parameter variable settings and their values, including the bus voltage *V*_bus_, support capacitor *C*_bus_, load inductor *L*, and switching frequency *f* in Figure 7.

## 4. Application of Gate-Voltage-Boosting Technology in IGBT Driver Circuit

### 4.1. Principle of Gate-Voltage-Boosting Technology

Without changing the traction inverter control strategy, changing the driving parameters of IGBT is the most effective way to reduce the loss of IGBT. In this paper, a related mathematical model is established by exploring the relationship between gate voltage and the loss of IGBT. An appropriate gate voltage is chosen to reduce IGBT losses.

**Figure 9:**
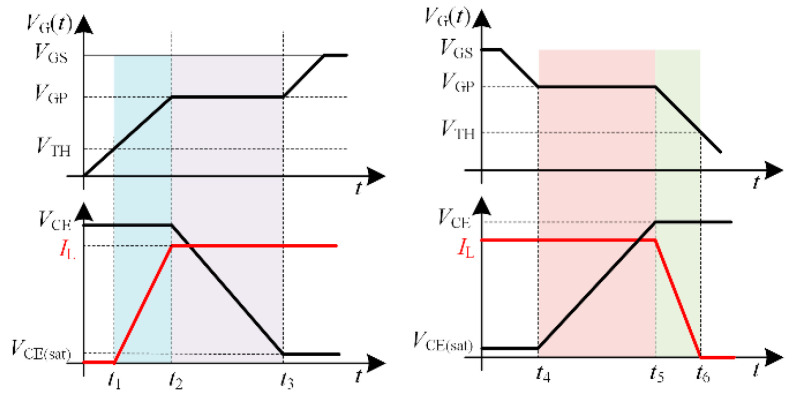
IGBT switching waveform.

The loss of IGBT is divided into switching loss and on-state loss. Firstly, the switching loss is analyzed. Figure 9 shows the typical turn-on and turn-off waveforms of IGBT. In order to simplify the analysis process, the waveforms are linearized.

The turn-on process of IGBT starts from the rise of collector current to the drop of collector-emitter voltage to the saturation voltage drop, that is, from *t*_1_ to *t*_3_. The turn-on energy of the IGBT is:

(15) $$E_{\mathrm{on}}=E_{t_{1}{-t}_{2}}+E_{t_{2}{-t}_{3}}$$

(16) $$E_{t_{1}{-t}_{2}}=\frac{1}{2}I_{L}(t_{2}-t_{1})V_{\mathrm{CE}}$$

(17) $$E_{t_{2}{-t}_{3}}=\frac{1}{2}V_{\mathrm{CE}}(t_{3}-t_{2})I_{L}$$

*E*_on_ is the turn-on energy of the IGBT, and *E*_t1−t2_ and *E*_t2−t3_ are the energy in *t*_1_−*t*_2_ and *t*_2_−*t*_3_. *V*_CE_ is the voltage across CE when the IGBT is turned on. *I*_L_ is the load current.

The time for the gate voltage to reach the threshold voltage is:

(18) $$t_{1}=R_{G}[C_{\mathrm{GE}}+C_{\mathrm{GC}}]\ln\left(\frac{V_{\mathrm{GE}}}{V_{\mathrm{GE}}-V_{\mathrm{TH}}}\right)$$

When the gate voltage reaches the Miller plateau voltage, the collector current rises to the load current *I*_L_, then:

(19) $$\begin{array}{ll} t_{2} & =R_{G}[C_{\mathrm{GE}}+C_{\mathrm{GC}}]\\ & \times \ln\left[\frac{V_{\mathrm{GE}}\mu_{\mathrm{ni}}C_{\mathrm{OX}}Z}{V_{\mathrm{GE}}\mu_{\mathrm{ni}}C_{\mathrm{OX}}Z-L_{\mathrm{CH}}\sqrt{I_{L}}-V_{\mathrm{TH}}\mu_{\mathrm{ni}}C_{\mathrm{OX}}Z}\right] \end{array}$$

Therefore, the duration of *t*_1_−*t*_2_ is:

(20) $$\begin{array}{ll} t_{2}-t_{1} & =R_{G}[C_{\mathrm{GE}}+C_{\mathrm{GC}}]\\ & \times \ln\left[1+\frac{L_{\mathrm{CH}}\sqrt{I_{L}}}{(V_{\mathrm{GE}}-V_{\mathrm{TH}})\mu_{\mathrm{ni}}C_{\mathrm{OX}}Z-L_{\mathrm{CH}}\sqrt{I_{L}}}\right] \end{array}$$

*R*_G_ is the gate resistor, *C*_GE_ is the gate capacitor, *C*_GC_ is the Miller capacitor, *L*_CH_ is the channel length, *V*_GE_ is the gate voltage, *V*_TH_ is the turn-on threshold voltage, *μ*_ni_ is the electron mobility, *C*_ox_ is the oxide capacitor, and *Z* is the channel width.

The duration of *t*_2_−*t*_3_ is:

(21) $$t_{3}-t_{2}=\frac{R_{G}C_{\mathrm{GC}}}{V_{\mathrm{GE}}-V_{\mathrm{GP}}}[V_{\mathrm{CE}}-V_{\mathrm{CE}(\mathrm{sat})}]$$

(22) $$V_{\mathrm{GP}}=V_{\mathrm{TH}}+\sqrt{\frac{I_{L}L_{\mathrm{CH}}}{\mu_{\mathrm{ni}}C_{\mathrm{OX}}Z}}$$

*V*_GP_ is the Miller platform voltage. Combined with (16)–(22), the IGBT turn-on energy is:

(23) $$\begin{array}{ll} E_{\mathrm{on}} & =\frac{1}{2}I_{L}V_{\mathrm{CE}}R_{G}[C_{\mathrm{GE}}+C_{\mathrm{GC}}]\\ & \times \ln\left[1+\frac{L_{\mathrm{CH}}\sqrt{I_{L}}}{(V_{\mathrm{GE}}-V_{\mathrm{TH}})\mu_{\mathrm{ni}}C_{\mathrm{OX}}Z-L_{\mathrm{CH}}\sqrt{I_{L}}}\right]\\ & +\frac{1}{2}I_{L}V_{\mathrm{CE}}\frac{R_{G}C_{\mathrm{GC}}}{V_{\mathrm{GE}}-V_{\mathrm{GP}}}[V_{\mathrm{CE}}-V_{\mathrm{CE}(\mathrm{sat})}] \end{array}$$

The turn-off process of IGBT is similar to the turn-on process. The turn-off energy of IGBT is:

(24) $$E_{\mathrm{off}}=E_{t_{4}{-t}_{5}}+E_{t_{5}{-t}_{6}}$$

The loss in *t*_4_−*t*_5_ and *t*_5_−*t*_6_ can be expressed as:

(25) $$E_{t_{4}{-t}_{5}}=\frac{1}{2}V_{\mathrm{CE}}(t_{5}-t_{4})I_{L}$$

(26) $$E_{t_{5}{-t}_{6}}=\frac{1}{2}I_{L}(t_{6}-t_{5})V_{\mathrm{CE}}$$

*E*_off_ is the turn-off energy of IGBT, and *E*_t4−t5_ and *E*_t5−t6_ are the energy in the time periods of *t*_4_−*t*_5_ and *t*_5_−*t*_6_.

The duration of *t*_4_−*t*_5_ is:

(27) $$t_{5}-t_{4}=R_{G}C_{\mathrm{GE}}\frac{V_{\mathrm{CE}}-V_{\mathrm{CE}(\mathrm{sat})}}{V_{\mathrm{GP}}}$$

The duration of *t*_5_−*t*_6_ is:

(28) $$t_{6}-t_{5}=R_{G}[C_{\mathrm{GE}}+C_{\mathrm{GC}}]\ln\left(\frac{V_{\mathrm{GP}}}{V_{\mathrm{TH}}}\right)$$

Combined with (24)–(28), the turn-off energy of IGBT is:

(29) $$\begin{array}{ll} E_{\mathrm{off}} & =\frac{1}{2}V_{\mathrm{CE}}I_{L}R_{G}C_{\mathrm{GE}}\frac{V_{\mathrm{CE}}-V_{\mathrm{CE}(\mathrm{sat})}}{V_{\mathrm{GP}}}\\ & +\frac{1}{2}V_{\mathrm{CE}}I_{L}R_{G}(C_{\mathrm{GE}}+C_{\mathrm{GC}})\ln\left(\frac{V_{\mathrm{GP}}}{V_{\mathrm{TH}}}\right) \end{array}$$

The switching loss of IGBT can be expressed as:

(30) $$P_{I\_s}=f_{\mathrm{sw}}(E_{\mathrm{on}}+E_{\mathrm{off}})$$

The turn-on energy of IGBT is negatively correlated with the gate voltage. Under the condition of constant switching frequency, the increase in gate voltage will lead to lower switching loss of IGBT.

In addition to the switching loss, the loss of IGBT also include the on-state loss, which can be expressed as:

(31) $$P_{I\_c}=V_{\mathrm{CE}(\mathrm{sat})}I_{L}D$$

Raising the IGBT’s gate voltage can lower the device’s on-state voltage drop and, as a result, lower the device’s on-state loss. The following is the impact mechanism:

When a large forward voltage is applied to the gate of IGBT, it operates in the on-state. The P-I-N rectifier and MOSFET are the two primary components of the IGBT’s on-state equivalent circuit. The formula below can be used to calculate the P-i-N junction’s voltage drop:

(32) $$V_{\mathrm{PiN}}=\frac{2kT}{q}\ln\left[\frac{J_{C}W_{N}}{4qD_{a}n_{i}F(W_{N}/2L_{a})}\right]$$

*k* is the Boltzmann constant, *T* is the temperature, *J*_C_ is the collector current density, *L*_a_ is the bipolar diffusion length, *W*_N_ is the N-type base width, *q* is the electronic charge, *F* is a function relation, and *D*_a_ is the bipolar diffusion coefficient.

The function F(*W*_N_/2*L*_a_) is related to the turn-on voltage drop for a P-i-N junction. The turn-on voltage loss is lowest when 2*L*_a_ = *W*_N_.

So, the IGBT on-state voltage drop is:

(33) $$\begin{array}{ll} V_{\mathrm{CE}(\mathrm{sat})} & =V_{\mathrm{PiN}}+V_{\mathrm{MOSFE}T}\\ & =\frac{2kT}{q}\ln\left[\frac{J_{C}W_{N}}{4qD_{a}n_{i}F(W_{N}/2L_{a})}\right]\\ & +(V_{\mathrm{GE}}-V_{\mathrm{TH}})[1-\sqrt{1-\frac{2pL_{\mathrm{CH}}J_{C}}{\mu_{\mathrm{ni}}C_{\mathrm{OX}}{\left(V_{\mathrm{GE}}-V_{\mathrm{TH}}\right)}^{2}}}] \end{array}$$

By combining the aforementioned formulae, it is shown that lowering the IGBT loss can be achieved by raising the gate voltage.

**Figure 10:**
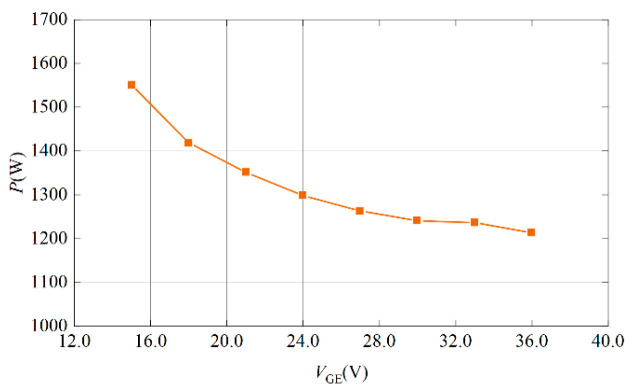
IGBT loss curve under different gate voltage.

Based on the above research mechanism, the loss of FD500R65KE3 under different gate voltages is simulated in PSpice, as shown in Figure 10. The loss of IGBT gradually decreases with the increase in gate voltage, but the rate of decrease is smaller and smaller. When the gate voltage increases to a certain extent, the effect on the loss is very small.

The gate voltage in the datasheet should not exceed ±20 V, which is not the gate breakdown voltage. Rather, it is a value determined by the manufacturer considering the lifetime and reliability of IGBT. If the voltage blocking capability of the gate oxide layer is considered, its maximum withstand value can reach 70 V. Therefore, raising the IGBT gate voltage above 20 V will not damage the device.

It can be seen from Figure 10 that the relationship between the IGBT gate voltage and the loss is monotonic. But, the increase in gate voltage is not the bigger the better. The closer to the gate maximum blocking voltage, the lower the reliability of the IGBT. In order to select an appropriate gate voltage, the objective function *J* is established. *J* can evaluate the effect of gate voltage on IGBT loss and reliability:

(34) $$J(V_{\mathrm{GE}})=\alpha \frac{P_{\mathrm{GE}}(V_{\mathrm{GE}})}{P_{15}}+\beta \frac{V_{\mathrm{GE}}}{V_{\max}}$$

*P*_GE_(*V*_GE_) and *P*_15_ are the loss when the gate voltage is *V*_GE_ and 15 V. *V*_max_ is the maximum voltage blocking capability of IGBT gate oxide layer. *P*_GE_(*V*_GE_)/*P*_15_ represents the influence of gate voltage on IGBT loss, and the smaller the value, the smaller the IGBT loss. *V*_GE_/*V*_max_ represents the influence of the gate voltage on the reliability of IGBT, and the smaller the value, the higher the reliability. *α* and *β* are weights, *α* + *β* = 1. The values of *α* and *β* are related to the working conditions. When more attention is paid to the reduction in IGBT loss, the value of *α* is larger and *β* is smaller. Conversely, the value of *β* is larger and the value of *α* is smaller.

**Figure 11:**
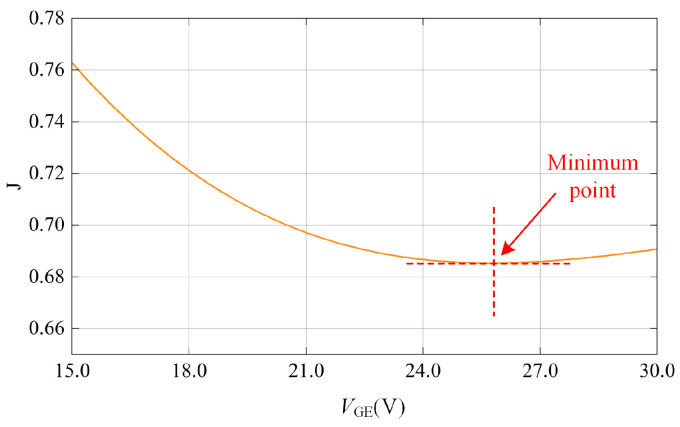
Influence curve of gate voltage on objective function.

The focus of this paper is to reduce the temperature fluctuation of IGBT. Therefore, more attention is paid to the effect of the gate voltage on the loss, and the *α* value is selected to be larger. Figure 11 shows the variation curve of the objective function *J* with the gate voltage when *α* = 0.7 and *β* = 0.3. The curve is not monotonic, and the minimum value is obtained when *V*_GE_ = 25.8 V. At this time, the working characteristics of IGBT device are optimal. In the actual design, the gate voltage of IGBT is usually selected as 26 V.

### 4.2. IGBT Driver Circuit Using Gate-Voltage-Boosting Technology

**Figure 12:**
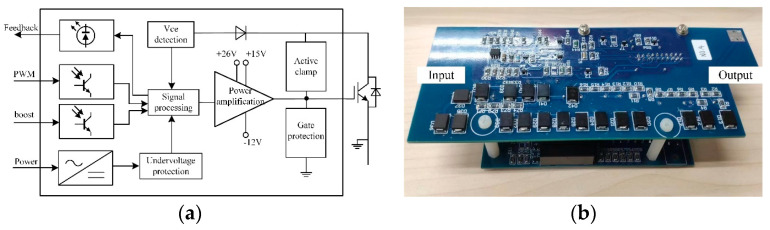
IGBT Driver (**a**) IGBT diver function block diagram, (**b**) IGBT driver physical diagram.

This work introduces a boost gate voltage circuit based on the features of typical IGBT drive circuits, such as under-voltage protection, active clamping, short-circuit protection, and gate protection. Under specific operating conditions, the circuit can increase the gate voltage from +15 V to +26 V, which will lower the IGBT loss. The function block diagram of the IGBT drive circuit with boost gate voltage is shown in Figure 12a, and the physical diagram is shown in Figure 12b. The driving circuit includes a signal input part, a power supply part, a protection part, a signal processing part, and a power amplifying part.

**Figure 13:**
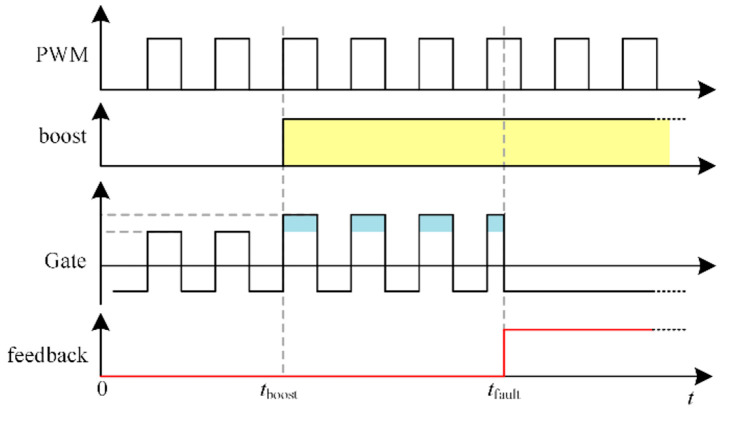
Timing diagram of the driver circuit.

PWM and boost signals are the two signals that make up the drive circuit’s signal input section. To ascertain whether the boost gate voltage circuit is operating, the two signals are combined. In order to make sure that the TCU is aware of whether the IGBT is malfunctioning, the drive circuit also incorporates a feedback function. Figure 13 displays the timing of the driving circuit. Upon a low signal, the driver circuit produces an output of +15 V/−12 V. At *t*_boost_, the boost signal becomes high and the gate boost circuit starts to work. +26 V/−12 V is the driving circuit’s output. IGBT has a short circuit fault at *t*_fault_. To turn off the IGBT, the driver circuit outputs a negative voltage right away.

**Figure 14:**
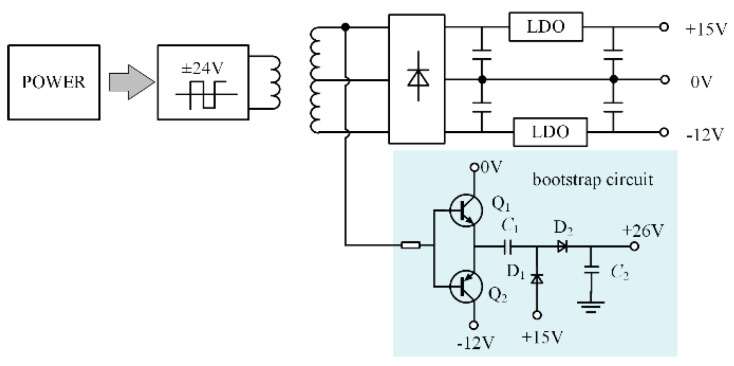
Bootstrap circuit.

External power pulses at a voltage/frequency of ±24 V/35 kHz are used to supply the drive power. Diode rectification and linear voltage regulator devices are then used to acquire the voltages of +15 V and −12 V. The power is designed to be 15 W, which satisfies the driving power requirements. The bootstrap circuit realizes 26 V, and Figure 14 displays the precise circuit schematic diagram.

The protection part of the drive circuit includes three parts: under-voltage protection, gate protection and short-circuit protection. The under-voltage protection is to monitor the output voltage of the driving power supply. Gate protection is to limit the gate voltage to ensure that the value of the short-circuit current is not too large. A bidirectional TVS diode is applied so that the gate voltage can be limited to not exceed the breakdown voltage of the TVS diode. The short-circuit protection is to quickly turn off the IGBT after a short-circuit of the IGBT, so that more serious failures can be prevented. In this design, *V*_CE (sat)_ desaturation detection method is used to realize short-circuit protection.

**Figure 15:**
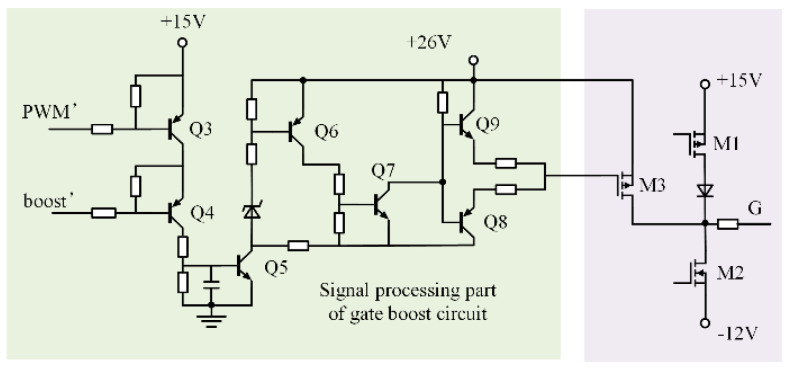
Signal processing part of gate boost circuit and power amplifier circuit.

The circuit diagram for the power amplifying and signal processing components of the gate boost circuit is displayed in Figure 15. The IGBT’s current capacity is increased when M3 is turned on because the gate voltage is pumped up to +26 V.

When the boost signal is low, the boost signal is high, Q4 is turned off, and Q5 is turned off. The gate boost circuit will not function, and the MOSFET M3 will not be turned on. +15 V/−12 V is the output of the drive circuit, which is controlled by the PWM signal. M3 is turned on when the boost and PWM signals are both high at the same time, resulting in a driving circuit output voltage of +26 V. A larger gate voltage is obtained by the IGBT, increasing its current capability.

PWM and boost signals are the two input signals used in the control portion of the gate boost circuit. Optocoupler isolation is required for these two signals as well as the driver circuit’s input signals (PWM and boost signals), which have a 180-degree phase difference.

## 5. Experimental Verification

**Figure 16:**
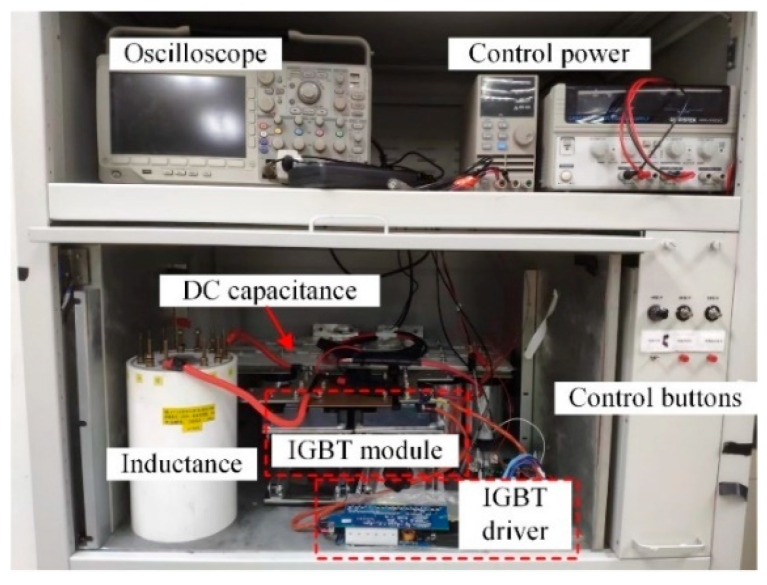
Double pulse test platform.

The experiment is finished using the double-pulse circuit, which confirms the logic of the intended IGBT driving circuit. In Figure 16, the test platform is displayed. Mitsubishi’s CM1200HC-66H IGBT module is chosen by the IGBT module. There is a load inductance of 100 uH and a DC bus voltage of 1800 V. The upper tube’s GE terminal is shorted in the double-pulse test to put it in a dependable shutdown state. The double pulse signal is sent to the lower tube. The load current increases to the predetermined value by varying the length of the first pulse width.

**Figure 17:**
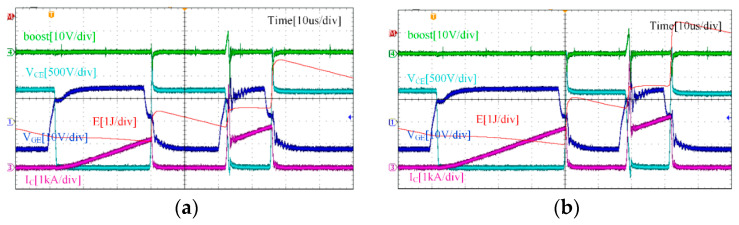
Traditional drive strategy: (**a**) Double-pulse waveform under rated condition, (**b**) Double-pulse waveform under overload condition.

**Figure 18:**
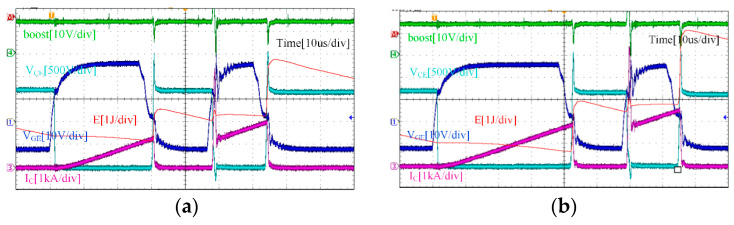
Boost drive strategy: (**a**) Double-pulse waveform under rated condition, (**b**) Double-pulse waveform under overload condition.

IGBT test circumstances are separated into two categories throughout the duration of the experiment: rated condition and overload condition. Under rated condition, the busbar voltage is 1800 V and the collector current is 1200 A. Under overload condition, the bus voltage remains at 1800 V, but the collector current becomes 1.5 times, which is 1800 A. As shown in Figure 17 and Figure 18, the double-pulse waveforms under the traditional driving strategy (+15 V gate voltage) and the boost driving strategy (+26 V gate voltage) are given. The drive circuit can sustain the dependability of the driver circuit and can raise the IGBT’s gate voltage by activating the boost signal, which confirms the dependability of the function of boosting the gate voltage, according to a comparison of the experimental waveforms.

Reducing the IGBT loss is the purpose of raising the gate voltage. Table 5 displays the calculated loss of IGBT under various driving methods and operational situations. In practice, the oscilloscope can track and record the *V*_CE_(*t*) and *I*_C_(*t*) values. The two can then be integrated using a mathematical function to obtain the energy of the IGBT, as shown by the red curves in Figure 17 and Figure 18.

**Figure 19:**
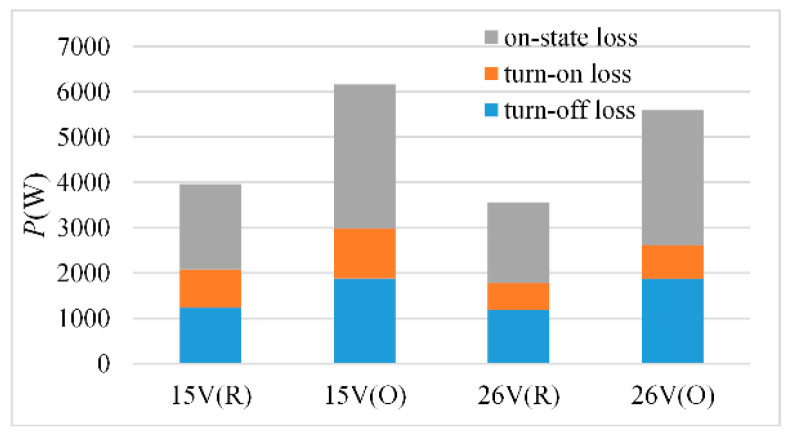
IGBT loss under different gate voltage.

When the working frequency of the IGBT is 800 Hz and the duty cycle is 50%, the loss calculation results are shown in Figure 19. O denotes an overload condition, while R denotes a rated condition.

The loss of IGBT is divided into three parts: turn-on loss, turn-off loss and on-state loss. The turn-on loss and on-state loss decrease as the gate voltage increases, and the turn-off loss remains unchanged, which is consistent with the previous theoretical analysis. As a result, increasing the gate voltage can effectively lower IGBT loss and increase IGBT lifetime.

## 6. Conclusions

The IGBT will experience an increase in current when the traction inverter is operating under overload conditions. The lifespan of IGBT is impacted by the significant fluctuations in the inverter’s temperature rise. This study considers the train’s emergency traction situation to be the overload condition. An analysis is conducted on the variations in the traction inverter’s output power and current. According to the modulation strategy and device working characteristics, the loss of IGBT under different working conditions are calculated. It is evident that during the emergency traction condition, the IGBT temperature varies significantly. Through theoretical analysis, the increase in IGBT gate turn-on voltage can reduce the loss of IGBT and reduce the fluctuation of temperature. An IGBT drive circuit with the function of boosting the gate voltage is designed without replacing the device. Finally, the function of the driving circuit is verified by experiments.

## Figures and Tables

**Table 1:** Train working parameters.

Parameters	Traction	Cruise
*I*_CP_ (A)	1592.5	112.9
*M*	0.9	0.9
η	90%	90%
cos *φ*	0.896	0.896
*f*_sw_ (HZ)	800	800
*V*_dc_ (V)	1800	1800

**Table 2:** IGBT module loss.

Parameters	Traction		Cruise	
	IGBT	Diode	IGBT	Diode
On-state loss (W)	1553.1	248.7	33.0	6.8
Switching loss (W)	993.3	287.5	265.8	67.4
Total loss (W)	2546.4	536.2	298.8	74.2
Total (W)	3082.6		373	

**Table 3:** IGBT module temperature rise.

Parameters	Traction	Cruise
*R*_th,jc_IGBT_ (K/kW)	8.5	
*R*_th,jc_Diode_ (K/kW)	17	
*R*_th,ch_ (K/kW)	6	
*R*_th,ha_ (K/kW)	2	
*T*_a_ (°C)	30	
*T*_jI_ (°C)	72.0	34.9
*T*_jD_ (°C)	43.4	31.9

**Table 4:** Simulation parameters.

Parameters	Value
*V*_bus_ (V)	3600
*C*_bus_ (F)	0.006
*L* (H)	0.0006
*f* (Hz)	800

**Table 5:** IGBT switching energy and on-state voltage.

Parameters	Value			
condition	15(R)	15(O)	26(R)	26(O)
*E*_on_ (J)	1.06	1.37	0.73	0.91
*E*_off_ (J)	1.54	2.35	1.49	2.34
*V*_CE(sat)_ (V)	3.14	3.54	2.98	3.32

## Data Availability

No new data were created.
